# PReventing Idiopathic SCOliosis PROgression (PRISCOPRO): A protocol for a quadruple-blinded, randomized controlled trial comparing 3D designed Boston brace to standard Boston brace

**DOI:** 10.1371/journal.pone.0255264

**Published:** 2021-08-09

**Authors:** Elias Diarbakerli, Anastasios Charalampidis, Allan Abbott, Paul Gerdhem

**Affiliations:** 1 Department of Clinical Science, Intervention and Technology, Karolinska Institutet, Stockholm, Sweden; 2 Department of Reconstructive Orthopaedics, Karolinska University Hospital, Stockholm, Sweden; 3 Department of Health, Medicine and Caring Sciences, Division of Prevention, Rehabilitation and Community Medicine, Unit of Physiotherapy, Linköping University, Linköping, Sweden; 4 Department of Orthopaedics, Linköping University Hospital, Linköping, Sweden; PLOS ONE, UNITED STATES

## Abstract

**Introduction:**

Idiopathic scoliosis is the most common spinal deformity in children. Treatment strategies aim to halt progression of the curve. Patients are treated mainly with thoracolumbosacral orthosis (TLSO) if indicated. This form of brace treatment has been shown to be cumbersome and tough on growing individuals. However, computer aided design and manufactured (CAD/CAM) braces might increase comfortability and ultimately outcome if compliance is improved. In a multicenter, randomized controlled trial, we aim to compare CAD/CAM designed Boston 3D-brace to standard Boston brace.

**Methods:**

Subjects: 170 previously untreated and skeletally immature children diagnosed with idiopathic scoliosis, aged 9–17 years of age (curve magnitude Cobb 25–40 degrees) will be included. Interventions: Both groups will receive a physical activity prescription according to the World Health Organization recommendations. Randomization will be performed 1:1 to a 3D CAD/CAM designed Boston 3D-brace or a standard Boston brace, both with prescribed daily wear time of 20 hours. Outcome: The subjects will participate in the study until curve progression or until skeletal maturity. The primary outcome variable is failure of treatment, defined as progression of the Cobb angle more than 6 degrees compared to the baseline x-ray. The progression is confirmed if seen on two consecutive standing spinal x-rays. Radiographs will be taken at each six-month follow-up. Secondary outcome measures include patient and clinical reported outcomes, including number of individuals requiring surgical intervention.

**Discussion:**

This study will show if efficacy in brace treatment can be improved with new brace designs.

**Trial registration:**

The protocol has been registered on ClinicalTrials.gov, identifier: NCT04805437.

## Introduction

Idiopathic scoliosis is the most common spinal deformity in children and adolescents with an estimated prevalence of 3% [1]. About one tenth of the children with scoliosis develop a deformity that requires treatment with brace or surgery with current treatment protocol. School screening programs in Sweden account for at least 200,000 children screened for scoliosis per year. Children with a suspected scoliosis are usually referred to orthopedic specialists for evaluation. Standard treatment consists of bracing 18–20 hours or more per day and gold standard in brace treatment is a rigid thoracolumbosacral orthosis (TLSO), aiming to halt curve progression by providing 3-dimensional (3D) correctional counterforces to the scoliosis curvature [2]. Previous literature on quality of life has suggested that brace treatment, being in many cases tough and cumbersome on adolescents, might have a negative impact on mental health and studies have also shown that patients surgically treated for idiopathic scoliosis are to a larger extent satisfied with management [3–5]. Outcomes of brace treatment depend to a large extent on wearing time [6]. Since many adolescents feel uncomfortable in the brace, it is of importance to combine efficacy and comfortability of the brace. In recent years, braces designed using computer-aided design and manufacturing system (CAD/CAM) have been developed, aiming to improve customization and comfortability for the patient. Using CAD/CAM might provide better 3D effect on the curvature rather than internal molded forms being inserted in the standard brace. Previous studies have shown promising results in producing lighter, more customized and efficient braces [7–10].

In a quadruple-blinded randomized controlled trial, we seek to compare CAD/CAM designed Boston 3D-brace to standard Boston brace in patients with moderate idiopathic scoliosis.

### The question at issue/hypothesis

We hypothesize that a CAD/CAM designed Boston 3D brace increases compliance and comfortability resulting in superior outcomes compared to the standard Boston brace.

## Methods

### Study design

**Fig 1:**
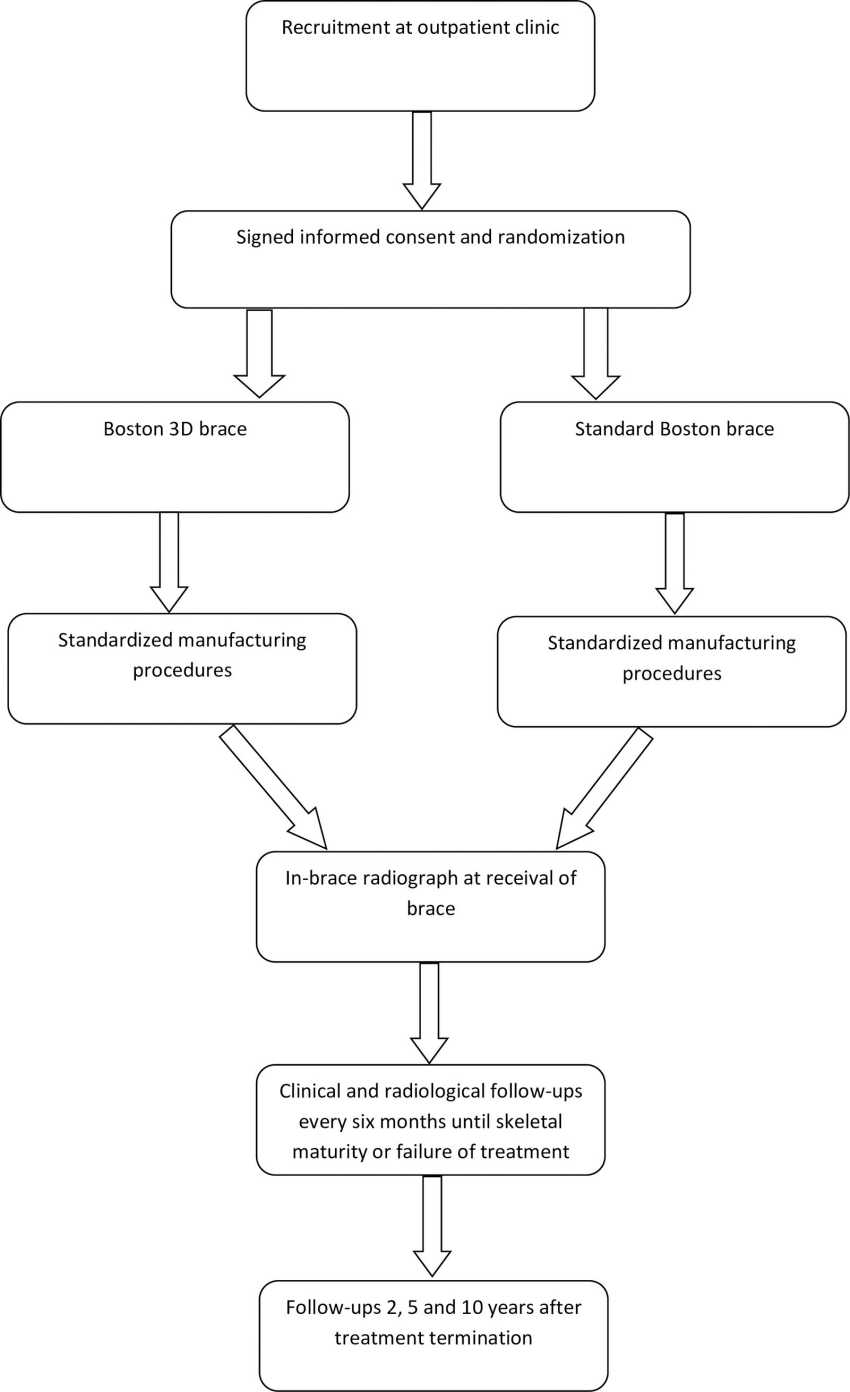
Flow scheme of patient recruitment and management.

This is a quadruple-blinded randomized controlled trial. The protocol has been registered on ClinicalTrials.gov, identifier: NCT04805437. Individuals who meet inclusion criteria will be asked for participation. After informed consent, randomization will take place. Currently, this study will be performed at Karolinska university hospital in Stockholm, Sweden. Further centers will be involved continuously. Fig 1 includes a flow scheme for the study.

**Fig 2:**
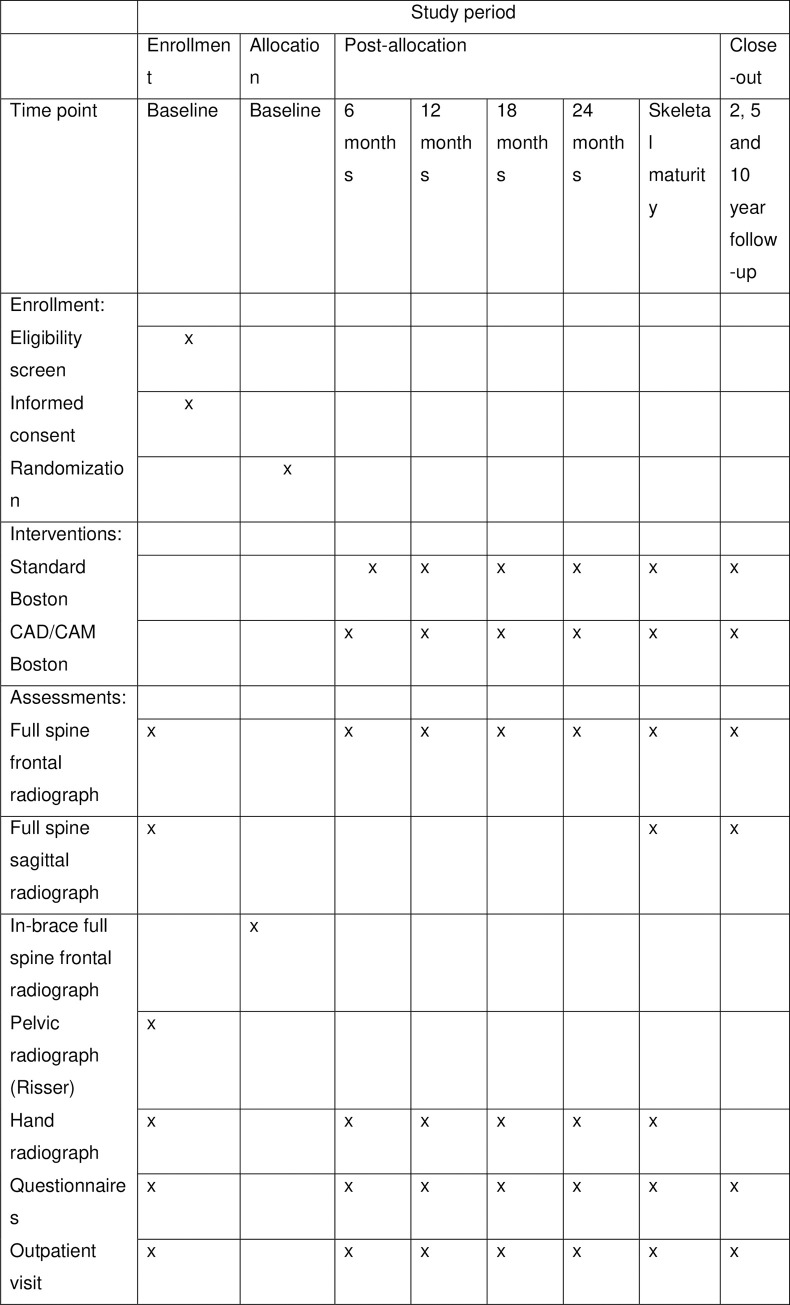
SPIRIT 2013 checklist: Recommended items to address in a clinical trial protocol and related documents.

Fig 2 illustrates SPIRIT checklist.

### Subjects

Patients referred to the participating clinics are consulted and examined by a health care provider specialized in spinal disorders. The magnitude of the scoliosis in the frontal plane is assessed by measuring the Cobb angle [11]. Upon inclusion, full spine frontal and sagittal radiographs, pelvic and hand radiographs will be taken. Up to three months old radiographs will be accepted. Prior or during the study, full spine Magnetic Resonance Imaging (MRI) may be done for all individuals at one occasion. The following inclusion criteria will be applied:

- Skeletally immature patients. Sanders score ≤ 6 [12], Risser sign ≤ 2 [13] and menarcheal status not more than one year for females.
- Age 9–17 years of age at inclusion.
- Primary curve of 25–40 degrees Cobb angle.
- Scoliosis of idiopathic nature with no previous brace or surgical treatment.
- Apex of the primary curve at T7 or caudal.

### Boston 3D brace

A CAD/CAM designed Boston 3D brace will be designed to the patient’s individual type of scoliosis. In-brace radiographs will be performed after prescription. Reinforcement of the intervention will be performed in conjunction with reassessment every six months. Patients are encouraged to use the brace for a minimum of 20 hours per day and to also continue with physical activities for the entirety of the study. Compliance will be monitored with a thermal sensor (iButton DS1922L Thermochron Data Logger, Measurement Systems Ltd, United Kingdom) built in the brace that measures wearing time. The thermal sensor will be set to measure every hour.

### Standard Boston brace

A standard Boston brace will be designed to the patient’s individual type of scoliosis. In brace radiographs will be performed after prescription. Reinforcement of the intervention will be performed in conjunction with reassessment every six months. Patients are encouraged to use the brace for a minimum of 20 hours per day and to also continue with physical activities for the entirety of the study. Compliance will be monitored with a thermal sensor (iButton DS1922L Thermochron Data Logger, Measurement Systems Ltd, United Kingdom) built in the brace that measures wearing time. The thermal sensor will be set to measure every hour.

### Non-participants

Patients not willing to undergo randomization will be treated in accordance with local procedures. Standard TLSO brace treatment will be offered. Non-participants will be asked to answer the same questionnaires as the study cohort at the time when treatment is initiated. The radiological and clinical result of these patients will be obtained from the regular orthopaedic files and radiographs taken.

### End of study

The study primary endpoint will be reached when the participant reaches skeletal maturity, defined as less than 1.0 cm growth of body height in six months, or if the curve progresses more than 6 degrees, compared to the baseline radiograph, seen on two consecutive spinal standing radiographs. Curve progression will not lead to change of brace. In the event that a Cobb angle surpasses 50 degrees, patients will be offered surgical treatment. Follow-up is planned to occur at 2, 5 and 10 years after skeletal maturity including standing full spine frontal and sagittal radiographs.

### Ethical permit

This study has been ethically approved by the Swedish Ethical Review Authority (approved 2021-04-20. Diary number: 2020–06502).

### Randomization procedure

After assessment, patients fulfilling the inclusion and exclusion criteria will be asked to give written and signed consent for participation. If the patient is aged 15 years or less, parental consent is also required. Patients who decline participation will be followed and monitored as part of clinical routine.

Randomization will be done in a 1:1 ratio. Randomization will be performed using an online module available through the Swedish spine register (www.swespine.se). Randomization sequence have been prepared by an independent data manager (www.medscinet.com), which runs the register platform, and is not accessible for the researchers.

### Blinding

Patients, care provider, investigators and outcome assessors will be blinded to the type of brace prescribed. Orthotist and research nurse will know what type of brace the participant receives but will not tell the participant which type he/she has received. The brace will not be brought inside the examination room during follow-ups. Readings of the thermal sensor will only be handled by the research nurse. For both patient groups, the procedure with scanning and measuring prior to bracing, and delivery of the brace, will be identical at the orthotist.

### Outcome measurements

Primary outcome measure is change in the Cobb angle of more than 6 degrees from baseline to the radiographic follow-ups and confirmed on two consecutive radiographs, similar to a previous randomized trial from our group [14]. Radiographs will be taken every six months in conjunction with clinical follow-ups until skeletal maturity. During each follow-up, two independent experts blinded to the type of intervention will assess the radiographs to determine if progression has occurred. If progression is suspected, additional radiographs may be taken for verification, also at additional time points not being considered as regular follow-ups.

Secondary outcome measures recorded at baseline and every six months for the entirety of the study include angle of trunk rotation, as measured with Bunnell’s scoliometer [15], patient-reported outcomes as measured with Scoliosis Research Society-22r, EQ-5D-youth version, Visual Analogue Scale-pain (VAS-pain), the International Physical Activity Questionnaire (IPAQ) short form and the pictorial part of Spinal Appearance Questionnaire (pSAQ) and hours in brace. Patients eventually also requiring surgical treatment will be recorded.

At each follow-up additional questions regarding protocol fulfillment (own-perceived compliance of the treatment), patient satisfaction and adverse effects (such as skin issues, pain and discomfort caused by bracing) will be monitored.

### Data integrity

Data will be entered by the participant directly in an online module, or by paper format and then entered by the research staff.

### Data analysis

Data from the trial will be compared based on the ’intention to treat’ (ITT) principle. An (ITT) analysis means that all patients, regardless of non-compliance, loss to follow-up or drop-out, remain in the analysis of the group to which they were randomized [16]. Multiple imputation or maximum likelihood methods of missing data will be used in the ITT analysis. A sensitivity analysis will be performed comparing the ITT data against a per-protocol data exclusively from patients who complied with the study protocol. If a patient fails treatment and is offered another treatment, such as surgery, the Cobb angle at the moment of commencing this treatment will be considered in the secondary outcome analysis of Cobb angle. Categorical parameters will be compared by the Chi-square test. Continuous and discrete parameters will be measured using parametric or non-parametric tests (depending on skewness) for group comparisons. Individual variables (such as actual brace time, in-brace correction and maturity) will be tested for their association with treatment effect by adding a predictor × treatment group interaction term to a regression equation. Additionally, Kaplan-Meier survival analyses will be used to display the probability of more than 6° Cobb progression over time for both trials.

### Power analysis

The end point failure of treatment is defined as an increase of the Cobb angle of at least 6° on two consecutive x-rays, when compared to the x-ray performed at time of inclusion. Based on a hypothesized failure rate of 2% in the 3D-brace group and 15% in the standard brace group [6] with 5% two-tailed significance level and 80% power and consideration for dropout of up to 20%, 85 individuals are required in each group.

## Discussion

This project on brace treatment for idiopathic scoliosis will determine efficacy of CAD/CAM designed Boston 3D brace compared to standard Boston brace in moderate idiopathic scoliosis. The study includes key methodological features in order to minimize bias in clinical trials such as true randomization, specification of eligibility criteria, blinding and intention-to-treat analysis. The methodological framework of this project is similar to a successfully managed randomized, but yet unpublished, trial from our group [14].

Our choice of outcomes and inclusion criteria is in line with recommendations for clinical trials studying idiopathic scoliosis. To date, there are many types of 3D designed braces under different labels. Our choice to compare standard Boston brace with Boston 3D brace is mainly due to the fact that these braces are more frequently used in Swedish healthcare compared to other brace designs. If CAD/CAM designed Boston 3D brace shows at least comparable outcomes to standard Boston brace, but better compliance and tolerability; future bracing may be less cumbersome on adolescents treated with brace.

Increasing comfortability and tolerability of brace treatment among adolescents with idiopathic scoliosis is of importance not only to avoid deterioration in quality of life but ultimately also to improve outcomes since few brace hours is a predictor of treatment failure [2, 17]. A previous literature review described difficulties in performing high-quality randomized trials on brace treatment mainly due to the fact that parents often reject their children to be randomly allocated to treatment [18]. The current study includes bracing treatment with identical dosing recommendations in both groups and therefore we believe that randomization will be less of an issue for participating families.

### Summary

This study has a quadruple-blinded, randomized controlled design including experienced healthcare providers in managing scoliosis patients. The study will investigate the efficacy of brace treatment in preventing progression of moderate idiopathic scoliosis. The novel findings will enable evidence-based recommendations as to the effect, comfortability and tolerability of brace treatment in idiopathic scoliosis.

## Supporting information

S1 Checklist SPIRIT checklist. (DOC)

S1 File World Health Organization (WHO) trial protocol. (PDF)

S2 File (DOCX)
