# Supplementary material for: PReventing Idiopathic SCOliosis PROgression (PRISCOPRO): A protocol for a quadruple-blinded, randomized controlled trial comparing 3D designed Boston brace to standard Boston brace
Source: PLoS One. 2021 Aug 9;16(8):e0255264. doi: 10.1371/journal.pone.0255264 (PMC8351964; doi:10.1371/journal.pone.0255264)
Supplement: S1 File — (PDF) [file pone.0255264.s002.pdf]

Scientific summary

Idiopathic scoliosis is the most common spinal deformity in children and adolescents with an estimated prevalence of 3% (1). About one tenth of the children with scoliosis develop a deformity that requires treatment with brace or surgery with the current treatment protocol. In Sweden, mild scoliosis curves not requiring treatment, but at risk for progression during childhood, are only observed until skeletal maturity without active treatment. If progression occurs and the scoliosis requires treatment, standard treatment consists of bracing 20 hours or more per day. Bracing has shown to have a large negative psychosocial impact on children and adolescents (2). Outcomes of brace treatment depend to a large extent on wearing time and since many adolescents feel uncomfortable in the brace, it is of importance to combine efficacy and comfortability of the brace. Scoliosis specific exercises have in previous studies shown possible benefits in mild scoliosis curves and may be used as a method to prevent brace treatment, but the findings are not generally accepted (3, 4). In two multicenter, randomized controlled trials, we seek to 1) compare scoliosis specific exercises to observation alone and 2) compare standard brace to a newly developed type of brace in a double-blinded and randomized fashion.

The question at issue/hypothesis

We hypothesize that:

-Scoliosis specific exercises reduce the number of children with scoliosis progressing to a severe deformity requiring brace treatment compared to observation alone.

-a newly developed brace increases compliance and comfortability resulting in superior outcomes compared to the standard brace.

Objectives and effects

The project aims to improve treatment methods for children and adolescents with idiopathic scoliosis.

In Sweden, children with mild scoliosis curves at risk for progression do not receive an active treatment. For children and their legal guardians, this can in many cases lead to frustration and anxiety. Furthermore, if brace treatment is necessary, it is a cumbersome and demanding treatment for adolescents and children in a vulnerable stage of life. If scoliosis specific exercises can be proven to reduce the number of cases requiring brace treatment, many children and adolescents with scoliosis can be spared from having brace treatment.

In the second trial, we wish to compare a newly developed brace to the standard brace of today. Since brace outcomes of scoliosis depend on compliance, the newly developed brace may improve outcomes by increasing compliance and comfortability. Such an effect might also lead to better tolerability and less psychological impact, leading to improved quality of life and outcomes.

Research field overview

School screening programs in Sweden account for at least 200,000 children screened for scoliosis. Children with a suspected scoliosis are usually referred to orthopedic specialists for evaluation. If a minor scoliosis exists, the child is only observed during growth. Moderate scoliosis is treated with brace, aiming to halt progression. Some international guidelines recommend scoliosis specific exercises in curves below 25°, however these guidelines are not universally accepted (5). With larger curves, bracing or surgical treatment are usually advocated (5, 6).

The role and efficacy of scoliosis specific exercises have been studied and studies have shown possible benefits. The literature suggests possible benefits for scoliosis patients when treated with scoliosis specific exercises, but the lack of evaluation and follow-up until skeletal maturity (7) and comparison the lack of comparison of scoliosis specific exercises with observation alone in mild scoliosis make valid conclusions difficult to draw (3). Therefore, there is a need of a randomized controlled trial addressing these issues.

Brace treatment for scoliosis is a tough treatment for children and adolescents at a vulnerable stage in life. The psychological impact is considerable, and in one study, 27% of the brace treated patients reported that the treatment had a major negative effect on their lives (2). Since scoliosis outcomes are to a large extent dependent on compliance (wearing at least 20 hours per day), it is crucial to have a brace that is rather comfortable and tolerable. Standard brace constructions correct mainly the curve in the coronal plane. Since scoliosis is a deformity in all three planes, new constructions for 3d-correction have been popular and shown promising results (8). However, a randomized controlled trial is needed to evaluate the effectiveness on quality of life, compliance, clinical and radiological outcomes of 3d-correction in comparison with the standard brace of today.

Project description

For the first study, patients referred to the participating clinics are consulted and examined by an orthopedic clinician specialized in spinal disorders. The magnitude of the scoliosis in the frontal plane is assessed by measuring the Cobb angle (9). For the first study, the following inclusion criteria will be applied:

- Skeletally immature patients. Sanders score ≤ 4 (10), Risser sign < 2 (ossification of the iliac crest) and no menarche for females.
- Age 9-15 years of age at inclusion.
- Primary curve of 15-24° according to Cobb.
- Scoliosis of idiopathic nature with no previous treatment.

For the second study, the following inclusion criteria will be applied:

- Skeletally immature patients. Sanders score ≤ 6, Risser sign ≤ 3 and menarche status not more than a year for females.
- Age 9-17 years of age at inclusion.
- Primary curve of 25-45 degrees according to Cobb.
- Scoliosis of idiopathic nature with no previous treatment.

As part of clinical routine, full spine Magnetic Resonance Imaging (MRI) can be used in order to exclude other pathology. Protocols will be published in clinicaltrials.gov.

Randomisation procedure

After assessment, patients fulfilling the inclusion and exclusion criteria will be asked to give written and signed consent for participation. If the patient is aged 15 years or less, parental consent is also required. Patients who decline participation will be followed as monitored as part of clinical routine.

Randomisation will be done in a 1:1 ratio. Randomisation will be performed using an online program available through the Swedish spine register. This platform is currently under development and will be ready in April 2020.

Blinding

In the first trial, blinding of patients and clinicians is not possible. However, for both studies, the randomisation status of the participants will not be disclosed to the two experts who will assess the patient's X-rays continuously throughout the study.

For the second trial, both the patients and the treating clinician will not know what type of brace the patient is having. For all patients, the procedure with scanning and measuring prior to bracing will be identical with the orthotist and time for delivery of the brace to the patient will be identical.

Data integrity

Data will be entered by the participant directly in an online module, or by paper format and then entered by the research staff. A minimum of 50% of the intervention quota is required to be fulfilled to be considered a compliant intervention.

Data analysis

Data from the trials will be compared based on the 'intention to treat' principle. An intention to treat (ITT) analysis means that all patients, regardless of non-compliance, loss to follow-up or drop-out, remain in the analysis of the group to which they were randomized (11). Multiple imputation of missing data will be used in the ITT analysis. A sensitivity analysis will be performed comparing the ITT data against a per-protocol data exclusively from patients who complied with the study protocol. If a patient in one trial fails treatment and is offered another treatment, such as full-time bracing or surgery, the Cobb angle at the moment of commencing this treatment will be considered in the secondary outcome analysis of Cobb angle. Categorical parameters will be compared by the Chi-square test. Continuous and discrete parameters will be measured using parametric or non-parametric tests (depending on skewness) for group comparisons. Individual variables will be tested for their association with treatment effect by adding a predictor × treatment group interaction term to a regression equation. Additionally, Kaplan-Meier survival analyses will be used to display the probability of more than 6° Cobb progression over time for both trials.

Outcome measurements

For both trials, primary outcome measures recorded at baseline and every six months for the entirety of the study is the Cobb angle until skeletal maturity when treatment is terminated. During each follow-up, two independent experts will assess the radiographs to determine if progression has occurred. The Cobb angle is formed by the inclination of the upper end plate of the upper end vertebra and the inclination of the lower end plate of the lower end vertebra measured on standard scoliosis x-ray images. The end point failure of treatment is defined as an increase of the Cobb angle of at least 6° from the time of the first x-ray on two consecutive x-rays, considered as the minimal clinically important difference (12). Additionally, for the first trial comparing scoliosis specific exercises to observation, the number of patients progressing to a curve requiring brace treatment will be recorded and compared in both groups. For the second study, patients eventually also requiring surgical treatment will be considered failures.

Secondary outcome measures recorded at baseline and every 6 months for the entirety of the study include angle of trunk rotation, as measured with Bunnell’s scoliometer (13), SRS-

22r and EQ-5D-Y quality of life questionnaires, VAS-pain, Spinal Appearance Questionnaire (SAQ) the International Physical Activity Questionnaire (IPAQ) short form, surgical rates, time in brace and time in training.

At each six month follow-up additional questions regarding protocol fulfillment, patient satisfaction and adverse effects will be asked for.

Power analysis

The end point failure of treatment is defined as an increase of the Cobb angle of at least 6° on two consecutive x-rays, when compared to the x-ray performed at time of inclusion. Based on a hypothesized failure rate of 10% in the scoliosis specific exercise group and 39% in the observation group, with a significance level of 5% and a power of 80%, consideration for dropout of up to 20% and an additional inclusion of five individuals per group, an estimated number of 45 individuals is required in each group. For the second trial, 85 individuals are required in each group based on the hypothesis of a 2% failure rate in the 3D-brace group and 15% in the standard brace group with 5% significance level, 80% power and consideration for dropout of up to 20%.

Interventions in the first trial

Scoliosis specific exercises

An active self-correction technique tailored to the type of curve will be applied with the aim to correct the scoliosis in the sagittal, coronal and horizontal plane. Patients will also be informed and educated in self-corrective behavior during task-oriented tasks in daily living in order to enhance neuromotor control of the spine and limbs. Training goals are directed towards postural control, spinal stability, muscular stabilization and endurance in corrective postures. Patients will have outpatient sessions once every two weeks the first 3 months and perform the exercises at home in 30-minutes sessions three times per week. Patients are encouraged to continue with non-specific self-mediated physical activities of moderate intensity at least 60 minutes daily, for the entirety of the study. Compliance will be monitored by using a mobile application where the patients record their sessions and can have contact with the research personnel.

Observational group (non-specific activity only)

Patients are encouraged to continue with non-specific self-mediated physical activities of moderate intensity at least 60 minutes daily, for the entirety of the study. A cognitive behavioral therapy approach to reinforce physical activity will be performed in conjunction

with reassessment every 6 months. Compliance will be monitored by using a mobile application where the patients record their sessions and can have contact with the research personnel.

Interventions in second trial

3-dimensional brace treatment

A 3-dimensional Boston brace will be designed to the patient’s individual type of scoliosis. A cognitive behavioral therapy approach to reinforcement brace use will be performed in conjunction with reassessment every 6 months. Patients are encouraged to use the brace for 20 hours per day and to also continue with physical activities for the entirety of the study. Compliance will be monitored with a heat sensor built in the brace that measures wearing time.

Standard brace

A standard Boston brace will be designed to the patient’s individual type of scoliosis. A cognitive behavioral therapy approach to reinforcement brace use will be performed in conjunction with reassessment every 6 months. Patients are encouraged to use the brace for 20 hours per day and to also continue with physical activities for the entirety of the study. Compliance will be monitored with a heat sensor built in the brace that measures wearing time.

Preliminary results

Our study group have published multiple peer reviewed papers in leading journals concerning spinal deformities. We have found that physical activity may be affected in the short term after brace treatment. Previous studies have indicated lower bone mineral density among individuals with scoliosis, but we have not seen a higher fracture risk in adults with scoliosis compared to controls. We have presented quality of life data on treated and untreated adults, thus many years after treatment and also compared quality of life in males and females with scoliosis. We have published normative data for the most commonly used scoliosis specific quality of life questionnaire which is an important basis for the description of quality of life during scoliosis treatment in the planned project. In collaboration with others, we have detected and confirmed some of the genes involved in the development of scoliosis. We have also seen a steep increase in the number of surgeries for idiopathic scoliosis during more than a decade and speculate that this could be due to the previous decline in brace treatment. We are finalizing a multicenter study with similar methodology to the current project, but no results yet exist.

Ethical issues

There is evidence for the use of a Boston brace but since this has evident drawbacks due to cumbersome treatment and psychological concerns, compliance is often poor. Scoliosis specific exercises might reduce the number of mild scoliosis cases progressing and requiring full-time bracing. Furthermore, cases requiring full-time bracing might benefit from a 3-dimensionally constructed brace which might increase comfortability and compliance.

Patient benefit/significance for the health service

Planned implementation

The findings will at study end be reported in research networks, at international conferences and as reports in peer-reviewed journals. Since scoliosis specific exercises are not used at all in Sweden, the results of the project might change treatment strategies for mild scoliosis. Furthermore, replacing the standard brace of today with a 3-dimensional brace can be implemented immediately after completion of the project.

Clinical advocacy

All research participants are recruited during regular health care visits. All collaborators are clinicians and experts in managing spinal deformities.

Generalizability of anticipated findings

This project can possibly change treatment strategies for Swedish scoliosis patients radically and may have a large effect on patients, health care system and society. It will show whether active treatment for mild scoliosis can reduce the need of bracing and whether bracing can be less cumbersome and tough on adolescents and children with scoliosis.

References

1. Willner S, Uden A. A prospective prevalence study of scoliosis in Southern Sweden. Acta orthopaedica Scandinavica. 1982;53(2):233-7.

2. Danielsson A. J, Wiklund I, Pehrsson K, Nachemson A. L. Health-related quality of life in patients with adolescent idiopathic scoliosis: a matched follow-up at least 20 years after treatment with brace or surgery. European spine journal. 2001;10(4):278-88.

3. Monticone M, Ambrosini E, Cazzaniga D, Rocca B, Ferrante S. Active self-correction and task-oriented exercises reduce spinal deformity and improve quality of life in subjects with mild adolescent idiopathic scoliosis. Results of a randomized controlled trial. European spine journal. 2014;23(6):1204-14.

4. Negrini S, Zaina F, Romano M, Negrini A, Parzini S. Specific exercises reduce brace prescription in adolescent idiopathic scoliosis: a prospective controlled cohort study with worst-case analysis. Journal of rehabilitation medicine. 2008;40(6):451-5.

5. Negrini S, Donzelli S, Aulisa A. G, Czaprowski D, Schreiber S, de Mauroy J. C, et al. 2016 SOSORT guidelines: orthopaedic and rehabilitation treatment of idiopathic scoliosis during growth. Scoliosis and spinal disorders. 2018;13:3.

6. Dolan L. A, Weinstein S. L. Surgical rates after observation and bracing for adolescent idiopathic scoliosis: an evidence-based review. Spine. 2007;32(19 Suppl):S91-S100.

7. Kuru T, Yeldan I, Dereli E. E, Ozdincler A. R, Dikici F, Colak I. The efficacy of three-dimensional Schroth exercises in adolescent idiopathic scoliosis: a randomized controlled clinical trial. Clinical rehabilitation. 2016;30(2):181-90.

8. Negrini S, Marchini G. Efficacy of the symmetric, patient-oriented, rigid, three-dimensional, active (SPoRT) concept of bracing for scoliosis: a prospective study of the Sforzesco versus Lyon brace. Eura Medicophys. 2007;43(2):171-81; discussion 83-4.

9. Cobb JR. Outline for the study of scoliosis. Am Acad Orthop Surg Instr Course Lect. 1948;5:261-75.

10. Sanders J. O, Khoury J. G, Kishan S, Browne R. H, Mooney J. F, 3rd, Arnold K. D, et al. Predicting scoliosis progression from skeletal maturity: a simplified classification during adolescence. The Journal of bone and joint surgery American volume. 2008;90(3):540-53.

11. Hollis S., Campbell F. What is meant by intention to treat analysis? Survey of published randomized controlled trials. BMJ. 1999;319(7211):670-4.

12. Negrini S, Minozzi S, Bettany-Saltikov J, Chockalingam N, Grivas T. B, Kotwicki T, et al. Braces for idiopathic scoliosis in adolescents. The Cochrane database of systematic reviews. 2015;6:CD006850.

13. Bunnell W. P. An objective criterion for scoliosis screening. The Journal of bone and joint surgery American volume. 1984;66(9):1381-7.
