# Supplementary material for: PReventing Idiopathic SCOliosis PROgression (PRISCOPRO): A protocol for a quadruple-blinded, randomized controlled trial comparing 3D designed Boston brace to standard Boston brace
Source: PLoS One. 2021 Aug 9;16(8):e0255264. doi: 10.1371/journal.pone.0255264 (PMC8351964; doi:10.1371/journal.pone.0255264)
Supplement: S2 File — (DOCX) [file pone.0255264.s003.docx]

1. **Primary Registry and Trial Identifying Number**
   ClinicalTrials.gov, identifier: NCT04805437.
2. **Date of Registration in Primary Registry**
   03/13/2021
3. **Secondary Identifying Numbers**
   -
4. **Source(s) of Monetary or Material Support**
   All material will be funded by the research group.
5. **Primary Sponsor**
   Karolinska Institutet
6. **Secondary Sponsor(s)**
   -
7. **Contact for Public Queries**
   [elias.diarbakerli@sll.se](mailto:elias.diarbakerli@sll.se), tel: +460851770000, adress: Karolinska university hospital, 171 64, Stockholm, Sweden
8. **Contact for Scientific Queries**
   Elias Diarbakerli, PT, PhD. [elias.diarbakerli@sll.se](mailto:elias.diarbakerli@sll.se), tel: +460851770000, adress: Karolinska university hospital, 171 64, Stockholm, Sweden
   The contact for scientific queries must include:
9. **Public Title**
   Brace Treatment for Idiopathic Scoliosis
10. **Scientific Title**
    PReventing Idiopathic SCOliosis PROgression (PRISCOPRO).
11. **Countries of Recruitment**
    Sweden
12. **Health Condition(s) or Problem(s) Studied**
    Idiopathic scoliosis
13. **Intervention(s)**
    Experimental: 3D TLSO

A 3-dimensional Boston brace will be designed to the patient's individual type of scoliosis. In-brace radiographs will be performed after prescription. Reinforcement of the assigned intervention will be performed in conjunction with reassessment every 6 months. Patients are encouraged to use the brace for 20 hours per day and to also continue with physical activities for the entirety of the study. Compliance will be monitored with a heat sensor built in the brace that measures wearing time.

Active Comparator: Standard TLSO

A standard Boston brace will be designed to the patient's individual type of scoliosis. In brace radiographs will be performed after prescription. Reinforcement of the assigned intervention will be performed in conjunction with reassessment every 6 months. Patients are encouraged to use the brace for 20 hours per day and to also continue with physical activities for the entirety of the study. Compliance will be monitored with a heat sensor built in the brace that measures wearing time.

**Key Inclusion and Exclusion Criteria**
Inclusion Criteria:

- Cobb 25-40 degrees
- Skeletally immature, Sanders score of 6 or less and Risser 2 or less.
- Menarche status maximum one year in females
- Aged 9-17 years
- No previous brace treatment or surgery for scoliosis
- Apex of the primary curve at T7 or caudal

Exclusion Criteria:

- Non-idiopathic scoliosis (i.e. neuromuscular, syndromic or congenital scoliosis)
- Previous spine surgery

1. **Study Type**
   Interventional study
   - Study design:
     - Randomized in an online module (www.swespine.se)
     - Quadruple-blinded (Participant, Care Provider, Investigator, Outcomes Assessor
     - Single arm assignment
2. **Date of First Enrollment**
   First participant enrolled 30^th^ of April 2021.
3. **Sample Size**
   170 individuals planned to be enrolled.
4. **Recruitment Status**
   Recruitment status of this trial:
   - Recruiting: participants are currently being recruited and enrolled
5. **Primary Outcome(s)**
   Primary outcome measure is change in the Cobb angle of more than 6 degrees from baseline to the radiographic follow-ups and confirmed on two consecutive radiographs, similar to a previous randomized trial from our group
6. **Key Secondary Outcomes**
   Secondary outcome measures recorded at baseline and every six months for the entirety of the study include angle of trunk rotation, as measured with Bunnell’s scoliometer. patient-reported outcomes as measured with Scoliosis Research Society-22r, EQ-5D-youth version, Visual Analogue Scale-pain (VAS-pain), the International Physical Activity Questionnaire (IPAQ) short form and the pictorial part of Spinal Appearance Questionnaire (pSAQ) and hours in brace. Patients eventually also requiring surgical treatment will be recorded.

At each follow-up additional questions regarding protocol fulfillment (own-perceived compliance of the treatment), patient satisfaction and adverse effects (such as skin issues, pain and discomfort caused by bracing) will be monitored.

1. **Ethics Review**
   Approved by the Swedish Ethical Review Authority (Diary number: 2020-06502).

Board Affiliation: Uppsala

Phone: +460104750800 Email: registrator@etikprovning.se

Address: BOX 2110, 75002, Uppsala, Sweden

1. **Completion date**
   Anticipated last patient finishing treatment: September 2027

Anticipated last patient follow-up: September 2037

**Summary Results**
-

1. **IPD sharing statement**

Plan to share IPD: Yes, IPD underlying published manuscripts will be accessible for other researchers. The investigators in charge will be responsible for reviewing access requests. Crude data, randomization procedures and intervention details can be shared with other researchers upon request.
