# Supplementary material for: PReventing Idiopathic SCOliosis PROgression (PRISCOPRO): A protocol for a quadruple-blinded, randomized controlled trial comparing 3D designed Boston brace to standard Boston brace
Source: PLoS One. 2021 Aug 9;16(8):e0255264. doi: 10.1371/journal.pone.0255264 (PMC8351964; doi:10.1371/journal.pone.0255264)
Supplement: Peer review history

# Editor report: Decision Letter 0

**DOI:** 10.1371/journal.pone.0255264.r001

**Authors:** Nancy Beam

14 Jul 2021

PReventing Idiopathic SCOliosis PROgression (PRISCOPRO) : a protocol for a quadruple-blinded, randomized controlled trial comparing 3D designed Boston brace to standard Boston brace

PONE-D-21-16829

Dear Dr. Diarbakerli,

We’re pleased to inform you that your manuscript has been judged scientifically suitable for publication and will be formally accepted for publication once it meets all outstanding technical requirements.

Within one week, you’ll receive an e-mail detailing the required amendments. When these have been addressed, you’ll receive a formal acceptance letter and your manuscript will be scheduled for publication.

An invoice for payment will follow shortly after the formal acceptance. To ensure an efficient process, please log into Editorial Manager at http://www.editorialmanager.com/pone/, click the 'Update My Information' link at the top of the page, and double check that your user information is up-to-date. If you have any billing related questions, please contact our Author Billing department directly at authorbilling@plos.org.

If your institution or institutions have a press office, please notify them about your upcoming paper to help maximize its impact. If they’ll be preparing press materials, please inform our press team as soon as possible -- no later than 48 hours after receiving the formal acceptance. Your manuscript will remain under strict press embargo until 2 pm Eastern Time on the date of publication. For more information, please contact onepress@plos.org.

Kind regards,

Nancy Beam, PhD

Staff Editor

PLOS ONE

Journal requirements:

1. Thank you for stating the following financial disclosure:

 [The funders had and will not have a role in study design, data collection and analysis, decision to publish, or preparation of the manuscript.].

1. Please provide an amended Funding Statement that declares *all* the funding or sources of support received during this specific study (whether external or internal to your organization) as detailed online in our guide for authors at http://journals.plos.org/plosone/s/submit-now. 

1. Please state what role the funders took in the study.  If any authors received a salary from any of your funders, please state which authors and which funder. If the funders had no role, please state: "The funders had no role in study design, data collection and analysis, decision to publish, or preparation of the manuscript."

Please send your amended statements by return email; we will change the online submission form on your behalf.

2. We noticed you have some minor occurrence of overlapping text with the following previous publication(s), which needs to be addressed:

- https://bmcmusculoskeletdisord.biomedcentral.com/articles/10.1186/1471-2474-14-261

- https://clinicaltrials.gov/ct2/show/NCT04805437

In your revision ensure you cite all your sources (including your own works), and quote or rephrase any duplicated text outside the methods section. Further consideration is dependent on these concerns being addressed.

# Editor report: Acceptance letter

**DOI:** 10.1371/journal.pone.0255264.r002

**Authors:** Nancy Beam

28 Jul 2021

PONE-D-21-16829 

PReventing Idiopathic SCOliosis PROgression (PRISCOPRO): a protocol for a quadruple-blinded, randomized controlled trial comparing 3D designed Boston brace to standard Boston brace 

Dear Dr. Diarbakerli:

I'm pleased to inform you that your manuscript has been deemed suitable for publication in PLOS ONE. Congratulations! Your manuscript is now with our production department. 

If your institution or institutions have a press office, please let them know about your upcoming paper now to help maximize its impact. If they'll be preparing press materials, please inform our press team within the next 48 hours. Your manuscript will remain under strict press embargo until 2 pm Eastern Time on the date of publication. For more information please contact onepress@plos.org.

If we can help with anything else, please email us at plosone@plos.org. 

Thank you for submitting your work to PLOS ONE and supporting open access. 

Kind regards, 

PLOS ONE Editorial Office Staff

on behalf of

Dr. Nancy Beam 

Staff Editor

PLOS ONE
